# Dry and wet approaches for genome-wide functional annotation of conventional and unconventional transcriptional activators

**DOI:** 10.1016/j.csbj.2016.06.004

**Published:** 2016-06-29

**Authors:** Elisabetta Levati, Sara Sartini, Simone Ottonello, Barbara Montanini

**Affiliations:** Biochemistry and Molecular Biology Unit, Laboratory of Functional Genomics and Protein Engineering, Department of Life Sciences, University of Parma, Parco Area delle Scienze 23/A, 43124 Parma, Italy

**Keywords:** Transcription factors, Moonlighting transcriptional activators, Transcriptional activator trap, Yeast/bacterial one-hybrid, Protein binding microarrays, HT-SELEX

## Abstract

Transcription factors (TFs) are master gene products that regulate gene expression in response to a variety of stimuli. They interact with DNA in a sequence-specific manner using a variety of DNA-binding domain (DBD) modules. This allows to properly position their second domain, called “effector domain”, to directly or indirectly recruit positively or negatively acting co-regulators including chromatin modifiers, thus modulating preinitiation complex formation as well as transcription elongation. At variance with the DBDs, which are comprised of well-defined and easily recognizable DNA binding motifs, effector domains are usually much less conserved and thus considerably more difficult to predict. Also not so easy to identify are the DNA-binding sites of TFs, especially on a genome-wide basis and in the case of overlapping binding regions. Another emerging issue, with many potential regulatory implications, is that of so-called “moonlighting” transcription factors, i.e., proteins with an annotated function unrelated to transcription and lacking any recognizable DBD or effector domain, that play a role in gene regulation as their second job. Starting from bioinformatic and experimental high-throughput tools for an unbiased, genome-wide identification and functional characterization of TFs (especially transcriptional activators), we describe both established (and usually well affordable) as well as newly developed platforms for DNA-binding site identification. Selected combinations of these search tools, some of which rely on next-generation sequencing approaches, allow delineating the entire repertoire of TFs and unconventional regulators encoded by the any sequenced genome.

## 1 Introduction

Transcription factors (TFs) coordinate many important biological processes ranging from cell cycle progression, cellular differentiation and development to intracellular metabolism and environmental adaptation [1], [2], [3], [4]. Several human diseases, including cancer, are caused by alteration of regulatory programs and TFs are overrepresented among oncogene products [5]. About one-third of human developmental disorders is attributed to dysfunctional TFs [6] and programmed variations in the activity and/or specificity of TFs have also been documented as a major source of phenotypic diversity and evolutionary adaptation in various organisms [7], [8], [9]. Indeed, an increased complexity of TF-dependent regulatory networks is considered as a major driver of the emergence of metazoan life [10], [11], [12], [13].

A distinguishing feature of typical (“conventional”) TFs, compared to other transcriptional regulatory proteins, is their ability to interact with DNA in a sequence-specific manner. In the vast majority of cases, DNA-binding is achieved by one, sometimes more, DNA-binding domains (DBDs) and TFs are classified into superclasses and families according to the structural relatedness of their DBDs [14]. This DBD-based classification allows grouping different TFs on a structural basis. However, since the different structural motifs associated to the DNA-binding domains likely arose independently, this DBD-based structural classification does not necessarily mirror phylogenetic classification. In some cases, the DNA-binding domain provides clues on TF function. For example, homeo-domain containing TFs are often associated with developmental processes, while interferon regulatory factor family DBDs (helix-turn-helix motif) are functionally linked with the immune response [15], and fungal GATA factors are typically involved in nitrogen metabolism [16]. There are also proteins that display sequence-specific DNA-binding activity without any recognizable (“standard”) DBD [17], [18], [19] and many orphan DBD types are likely to be still discovered and structurally classified. In addition to the DBD itself, other regions can contribute to, and influence, DNA-binding activity; for example, DBD-flanking regions directly involved in TF dimerization and function (e.g. [20]).

So-called “effector domains” are the other essential components of TFs. They mediate gene activation or repression by promoting the formation of active or repressed chromatin states, by directly or indirectly recruiting positively or negatively acting co-regulators (co-activators and co-repressors), or by modulating preinitiation complex formation or productive transcription elongation. At variance with the DBDs, effector domains are much less conserved and thus considerably more difficult to identify simply on a sequence similarity basis.

As a prototypical characteristics of TFs, sequence-specific DNA-binding is the main and first feature that is commonly addressed while trying to characterize (or discover) a new TF. The DNA-binding specificity of a TF, i.e., its ability to discriminate between different sequence motifs, is only one of several factors that can contribute to determine the sites it actually binds in the genome. In fact, DNA-binding site occupancy can also be influenced by site accessibility in a chromatin context, by cooperation or competition with other sequence-specific DNA-binding proteins, and by interaction with histones and other architectural proteins and chromatin modifiers as well. Circumstantial evidence in favor of this added layer of complexity is represented by the fact that most eukaryotic TFs tend to recognize short and degenerate DNA sequence motifs, as opposed to the larger motifs preferred by prokaryotic TFs [21].

Characterization of intrinsic sequence binding preference (i.e., the one referring to a simplified and restricted TF-DNA interaction) ideally requires either in vitro or heterologous assay systems allowing overcoming potential confounding effects caused by other modulating or competing TFs. Recent technological advances have greatly increased the speed and reliability with which (semi)quantitative estimates of DNA-binding ability and specificity can be obtained. These include a range of methods — e.g., microarray-based approaches [19], [22], [23], [24], [25], [26], [27], [28], [29] as well as high-throughput (HT) sequencing-based approaches [30], [31], [32], [33], [34], [35], microfluidics-based technologies [36] and cell-based selection systems, also coupled with HT sequencing [37], [38], [39], [40] aimed at increasing the number of DNA sequences that can be interrogated in parallel (outlined in Table 1).

Another emerging issue is that of so-called “moonlighting” transcription factors, i.e., proteins with an official function unrelated to transcription that play a role in gene regulation as either activators or repressors, as their second job. Cases of moonlighting (“unconventional”) TFs, which are usually impossible to predict and particularly difficult to identify, have been documented in a variety of organisms ranging from bacteria to humans. For example, metabolic enzymes that moonlight as transcription factors, specifically designated as “trigger enzymes” or “metabolism-related transcription factors”, which include enzymes directly or indirectly involved in gene expression regulation, with different documented or purported roles such as DNA/RNA binding, modulatory interaction with selected transcription machinery components, co-activator/repressor function and chromatin remodeling [18], [19], [41], [42], [43].

Here we present a general overview of the approaches, including both well-established as well as newly developed high-tech strategies, currently utilized for the functional analysis of TFs, highlighting their advantages and potential limitations. Particular emphasis is placed on genome-scale experimental methods that are accessible even to non-highly specialized molecular biology laboratories. Untargeted methods, also suitable for the large-scale identification of unconventional transcription factors, i.e., putative TFs lacking any recognizable DBD, are also discussed.

## 2 Delineating the transcription factor repertoire at the genomic level

Following genome sequencing, the first step in the identification and functional characterization of the transcription factor repertoire of a newly sequenced organism is the classification of the entire TF catalog based on the presence of conserved DBDs. The potential involvement of individual TFs in specific cellular processes can also be investigated based on sequence similarity with previously characterized transcription factors. TF functional validation can be then pursued with the use of a genome-wide approach such as the transcriptional activator trap (TAT) assay, which relies on the heterologous expression of cDNA libraries or specific TF subsets in the yeast *Saccharomyces cerevisiae*. This method allows the rapid characterization of the transcriptional activation capabilities of predicted TFs. Because of its untargeted nature, the TAT assay also allows the identification of new putative unconventional activators lacking any recognizable DBD, which escape detection by search methods strictly based on sequence similarity.

### 2.1 TF identification and classification

Sequence-specific TFs are thought to comprise between 0.5 and 8% of the eukaryotic gene content and can be classified into superclasses and classes according to the structure of their DBDs [14], [44]. DBDs display a wide range of structural motifs encompassing a diverse array of protein folds, each representing a different solution to the problem of sequence-specific DNA recognition. More than 80 and 60 different DBD types have been recognized to-date in eukaryotes and prokaryotes, respectively, with very few DBD types shared between these two lineages and a few apparently lineage-specific DBD types. Several databases of experimentally and computationally identified transcription factors are available. Most of them are dedicated to specific phylogenetic groups such as the FlyTF [45], the Fungal FTFD [46], the mouse and human TFCat [17], and the bacterial RegulonDB [47] databases, while the “DNA-Binding Domain Database” includes more than 1000 completely sequenced genomes from multiple organisms [48] (see also [49] for a recent methodological review).

Putative TF-coding genes can be determined by computational approaches, the most sensitive and reliable of which is based on the genome-wide search of DBD-containing gene products using profile-based methods. Publicly available bioinformatic resources such as InterPro, Pfam, and SUPERFAMILY provide curated Hidden Markov Models (HMM) describing the amino acid sequences of groups of conserved polypeptide regions and domains. The “DNA-Binding Domain Database” provides, instead, a list of all Pfam and SUPERFAMILY DBD accession numbers. Conserved domain searches against known motifs can be comprehensively performed with the Blast2GO software [50], which allows scanning any deduced proteome against various databases available at the InterPro resource provided by the European Bioinformatics Institute (EBI) (http://www.ebi.ac.uk/Tools/InterProScan/) [51]. A genome-wide HMM search will return a set of genes coding for potential DBD-containing TFs. Some DBDs and their sequence models, however, may be promiscuous and produce false-positive hits to non-TF proteins that nonetheless bind DNA. For example, protein constituents of the core initiation complex, which should be removed, even if containing a DBD. Special attention should be paid to Cys_2_-His_2_ zinc-finger domains, which are not exclusively present in sequence-specific TFs, as well as to other proteins such as chromatin modifier proteins containing MYB/SANT, ARID, and HMG domains, which often lack intrinsic DNA-binding specificity [52], [53], [54]. Removal of inappropriate (“false-positive”) hits is aided by the Blast2GO software, thanks to the information it provides on the specific function of the proteins encoded by similar sequences (BLAST-based approach) [50]. Proteins containing structural features indicative of a non-nuclear localization, such as transmembrane domains, have to be removed as well.

This filtration step strongly depends on the specific content of the reference database and the ability of the search algorithms to detect the above domains. The final outcome of this search step, is a catalog of TFs classified according to their DBDs.

### 2.2 TF search based on sequence similarity

When possible, the results of the HMM search are integrated with the information derived from experimentally verified TFs. This step is important for at least two reasons. On one hand, even though the features of the DNA-binding domain may occasionally hint at the involvement in a specific process, the best way to infer TF function is based on sequence similarity. The other reason is that a HMM search may miss TFs bearing a DBD with a score below the significance threshold as well as TFs lacking a conventional DBD [18], [19]. Therefore, known non-standard TFs should also be included in this similarity search. A list of functionally-verified TFs can be retrieved from model organism-specific TF databases and/or from a dedicated literature search. A common method is to use pair-wise local sequence-alignment algorithms such as BLAST [55] to identify homologs of known TFs. Proteins sharing a high sequence similarity also extended to extra-DBD regions are likely to share the same function. Due to the structural properties of TFs, orthologous relationship among these proteins should be carefully verified by molecular phylogenetic inference, and not merely rely on a “best bidirectional hit” criterion. In fact, at variance with BLAST-based TF searches, which may be strongly biased by the presence of the highly conserved DBD, phylogenetic analyses are based on alignment of the entire sequence. A possible alternative to this quite laborious phylogenetic approach is to use a “best bidirectional hit” approach starting from TF sequences whose DBD has been masked [56].

Another word of caution regards the fact that not all TF homologs necessarily retain a similar function. In fact, TFs are among the most duplicated genes, and their function relies on different types of interactions, including protein-DNA interactions with specific genomic regulatory elements but also protein–protein interactions with other TFs and co-regulators. Moreover, TF-coding genes display a high degree of plasticity and tend to be under a stronger positive evolutionary selection compared to other genes (e.g., genes coding for metabolic enzymes as well as transport proteins and translation factors) [7], [8]. The only exception is the usually high conservation displayed by development-related TFs [9]. The initial outcome of gene duplication is the formation of two identical paralogs, which subsequently diverge through mutation, with a loss or gain of biomolecular interactions. By comparing the rates at which protein–protein and protein-DNA interactions are rewired, Reece-Hoyes et al. found that upstream regulatory regions are highly plastic and rapidly diverge, while the DNA sequence specificities of TFs are more stable over evolutionary time [57]. Further support to the notion that the DNA sequence specificity of TFs is generally more stable over evolutionary time was provided by Weirauch et al. [58]. By analyzing the DNA-binding preferences of over 1000 TFs from 131 different eukaryotes, these authors found that closely related DBDs (encompassing more than 50 different classes) always display similar DNA sequence preferences, thus paving the way to the identification of a TF/DBD “recognition code” [58].

### 2.3 Functional validation of TFs

At variance with the DNA-binding sites, which have been extensively investigated both functionally and structurally, the TF activation domains (AD) are poorly characterized from a structural point of view and much more difficult to predict. Pioneering work identified peculiar sequence features of eukaryotic ADs in the form of acidic regions bearing very few or no positively charged amino acids and displaying a net negative charge ranging from one to ten. An additional feature identified by these authors is the presence of small-sized hydrophobic amino acid patches interspersed with hydrophilic residues, leading to a predicted structure made by acidic residues-bearing β-turns and hydrophobic α-helices [59].

**Fig. 1:**
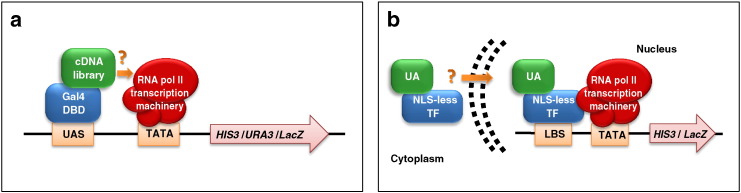
Identification and functional validation of TFs and unconventional activators. a. Schematic representation of the transcriptional activator trap (TAT) approach, as applied to the identification and functional validation of AD-containing, conventional and unconventional transcriptional activators. Reporter gene expression (*HIS3*, *URA3* and *LacZ*) is activated if the query TF (a selected subset or a whole cDNA library; *green*) fused to the Gal4-DBD (*blue*) behaves as a transcriptional activator — i.e., it is capable of recruiting RNA Pol II transcription machinery (*red*). UAS: upstream activating sequence (Gal4 DNA-binding site); TATA: TATA box. b. Nuclear transportation trap (NTT) assay used to test the autonomous nuclear localization capacity of putative unconventional activators. A chimeric protein (NLS-less TF, *blue*) comprising a modified bacterial DBD (LexA), a portion of the *E. coli* maltose binding protein and the yeast Gal4 AD, but lacking a nuclear localization signal (NLS), is fused to a candidate unconventional activator (UA, *green*). If the latter contains a NLS (either recognizable in silico or cryptic), it will direct the chimeric protein to the nucleus, thus leading to reporter gene (*HIS3*, *LacZ*) activation. The transcriptional machinery is in *red*. LBS: LexA binding site; TATA: TATA box.

Given the lack of a reliable in silico method for AD prediction, a relatively high-throughput functional analysis was developed in order to streamline, corroborate and extend TF-AD prediction. This untargeted search procedure leverages on the distinct and independent functions played by the two TF domains. It relies on a modified version of the yeast two-hybrid system, named transcriptional activator trap (TAT) assay, in which selected open reading frames or an entire cDNA library representative of the proteome of the organism of interest are fused to the DBD of the yeast TF Gal4 (Fig. 1a). The resulting fusion proteins are expressed in yeast and if the query sequence(s) code(s) for a transcriptional activator, the expression of Gal4-dependent reporter genes is activated and readily detected [60].

In its original application, the TAT assay was used to systematically test 6000 yeast proteins for transcriptional activator capacity and led to the identification of 451 transcriptional activators. Many of these activators were well-characterized transcriptional regulators or nuclear proteins but some of them corresponded to proteins without a prior record of transcriptional activation function [60]. The TAT-screen can also be employed as a high-throughput TF search approach using yeast as a heterologous system for identifying TFs encoded by a different, less experimentally tractable organism. For example, it was successfully employed for the identification of plant cDNAs coding for true transcription factors and previously unknown proteins endowed with the same activity [61]. It also allowed the validation of about one-fifth of the in silico predicted TFs from the mycorrhizal fungus *Tuber melanosporum* as well as the de novo identification of novel “putative unconventional activators” lacking a recognizable DBD and without any prior record of TF activity [56].

To investigate the gene transactivation capacity of plant ERF transcription factors under homologous conditions, a TAT assay was developed also for plant cells [62]. A chimeric construct in which the TF of interest is fused to a heterologous DNA-binding domain, such as the Gal4-DBD, was created and inserted into an expression (“effector”) vector, which was then used to transfect tobacco protoplasts along with a reporter gene plasmid bearing a luciferase gene under the control of Gal4-dependent upstream activating sequence (UAS) [62], [63]. By coupling a Gal4-UAS (bound by the chimeric transcriptional activator) with a plant repressor DNA-binding site, this system also allowed studying the effect of plant transcriptional repressors (ERFs) on reporter gene expression. As expected, reporter gene expression, and associated luminescence signal, decreased in protoplasts expressing both a transcriptional activator and a ERF repressor [62].

A TAT assay was also developed in mammalian cells, using a cDNA library cloned into a retroviral expression vector, in frame with a sequence coding for the yeast Gal4-DBD. The resulting library was packaged into retroviral particles, which were then delivered to a murine cell line harboring a Gal4-UAS-dependent, enhanced green fluorescent protein (*EGFP*) reporter gene, followed by FACS-assisted sorting of EGFP-positive cells. In this way, both known TFs as well as proteins whose TF activity had not been described before were isolated and functionally validated [64].

Despite the effectiveness and high-throughput potential of the heterologous yeast-TAT assay, both false-negative and false-positive results can be expected. As pointed out by Titz et al. [60], there is, in fact, an estimated false-negative rate of approximately 60%, due to the possibility that some proteins annotated as transcriptional activators do not behave as such in this assay. Possible reasons are the requirement for cofactors or specific nuclear conditions not available in *S. cerevisiae* and possible inhibition/alteration of the transcriptional activator function caused by the DBD fusion. Both may be solved, with some loss in throughput and ease of experimental manipulation, by switching to a homologous host. False-positive, instead, mainly results from the forced nuclear internalization of otherwise cytoplasmic proteins, imposed by the nuclear localization signal (NLS) associated with the Gal4-DBD. It should be noted, however, that many instances of apparently cytoplasmic proteins and/or DBD-lacking proteins capable of autonomously entering the nucleus and activating transcription (here designated as “unconventional activators”) are being increasingly reported. For example, various metabolic enzymes and other proteins as well, without a recognizable DBD have been found to be capable of entering the nucleus and activating reporter gene transcription [18], [19], [65]. Therefore, it is important that any comprehensive, genome-wide TF study extends beyond the predefined range of easily predictable DNA-binding proteins in order to provide new (and unbiased) insights for regulatory network analysis.

Given the NLS bias of the TAT assay it is essential to verify the autonomous nuclear entry ability of newly identified unconventional activators. In addition to various NLS search programs (e.g., cNLS Mapper [66], NLStradamus [67]), a specific yeast selection system, named “nuclear transportation trap” (NTT), has been developed for this purpose [68]. In the NTT, an artificial, NLS-lacking transcription factor, is fused to the query protein of interest. If the latter protein contains a functional NLS, it will redirect the chimeric TF to the nucleus, thus enabling transcriptional activation of reporter genes. The specificity of the NTT assay has subsequently been improved with the use of a single-copy, centromeric yeast vector and by fusing a portion of the *Escherichia coli* maltose binding protein to the chimeric TF, to avoid passive diffusion into the nucleus [69](Fig. 1b). Since the nuclear import apparatus is conserved between yeast and higher eukaryotes, the NTT assay can allow to functionally detect the presence of canonical as well as “cryptic” NLSs in any protein of interest regardless of its origin. For example, an NTT analysis revealed the presence of a canonical monopartite NLS and two unconventional (“cryptic”) NLSs in the viral transcriptional activator E1A from all six human adenovirus types [70].

Proper combination of the TAT and the NTT assays can thus represent an extremely informative first step toward the discovery and further characterization of proteins that, despite lacking any recognizable DBD, are capable of entering the nucleus and activating gene transcription (Levati et al. [65] describes an example of unconventional activators identified using this strategy).

## 3 Transcription factor DNA-binding site identification

Since DNA-binding is a key mechanistic feature of most (especially conventional) transcription factors, including general TFs and many activators, it is important to map the corresponding binding sites on the DNA (e.g., basal promoter-related sequences, distal activation sequences and enhancers). This is even more true, if one considers the steadily growing list of available wholly sequenced genomes and the vast amounts of associated gene expression data. Even though a general recognition “code” relating DBD amino-acid sequence to DNA-binding site specificity of TFs has not been worked out yet, many efforts are being made toward this goal [58]. Promoter and other *cis*-acting sequences can be inferred from multiple sequence alignments of regulatory sequences of ortholog genes and/or from the identification of conserved regulatory elements of genes sharing similar expression profiles and thus likely involved in similar biological processes [71].

Other, more direct, in vivo and in vitro approaches to study DNA-protein interaction and TF DNA-binding specificity are available and are briefly discussed below. Perhaps the most popular approach for in vivo TF DNA-binding site analysis is chromatin immunoprecipitation (ChIP) and related methods such as ChIP-chip and ChIP-seq [72], [73], [74]. It should be noted, however, that the DNA-binding sites retrieved from these assays, whose first step is performed in intact whole nuclei, may include the unpredictable contribution of specific co-regulators, local chromatin structure and other complex context effects [75], [76], [77], [78]. More focused information on intrinsic DNA-binding specificity can perhaps be derived from in vitro and heterologous high-throughput approaches (also reviewed in [79], [80], [81]). These can be paralleled and made more insightful by computational models such as position weight matrices (PWMs) that are employed to describe the DNA sequence specificity of a TF and to scan DNA sequences for the identification of potential DNA-binding sites.

### 3.1 DNA-centered approaches

Following the discovery of a regulatory region, it is important to identify the TFs that bind to it. Regulatory elements can be simple and relatively short sequences that can be analyzed in either a single-copy or a tandemly repeated form in order to increase the number of available binding sites. In other cases, more complex regulatory elements (e.g., an entire promoter region) can be analyzed in order to define the full repertoire of interacting TFs, so to attain a comprehensive view of the regulation of a gene of interest. The two most common DNA-centered approaches are perhaps the yeast one hybrid (Y1H) and the protein arrays.

**Fig. 2:**
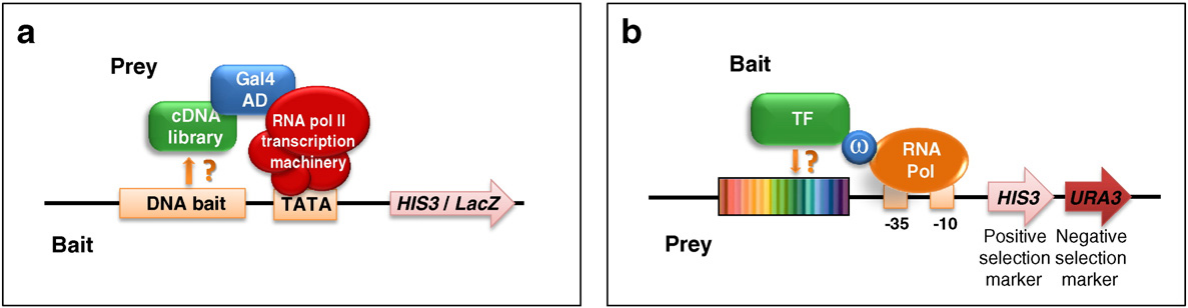
Heterologous in vivo approaches for TF DNA binding site identification. a. The yeast one-hybrid (Y1H) is a DNA-centered approach used to identify TFs capable of binding to a specific DNA element. The DNA sequence to be interrogated (“DNA bait”) is cloned into a selectable yeast plasmid, upstream of reporter genes such as *HIS3* and *LacZ*, and subsequently integrated into a mutated marker locus within the yeast genome. A TF of interest (either a selected one or a whole cDNA library; *green*) is expressed as a fusion with the yeast Gal4 activation domain (Gal4 AD, shown in *blue*). Positive hits (i.e., TFs bearing a DBD capable of interacting with the bait sequence) activate reporter gene expression. The transcriptional machinery is in *red*. TATA: TATA box. b. The bacterial one-hybrid (B1H) is a TF-centered approach used to identify the DNA element bound by a (putative) TF or activator. A bi-cistronic vector bearing a randomized region (*rainbowed*) upstream of two reporter genes (*HIS3* and *URA3*) is used as a “prey” to identify the DNA elements bound by the “bait” TF (or putative activator) (shown in *green*) fused to the ω subunit (*blue*) of bacterial RNA polymerase (*orange*). The yeast *URA3* gene is used as negative selection marker (5-FOA counter-selection) to eliminate self-activating DNA elements; the yeast *HIS3* gene is used as a positive selection marker to identify the DNA elements bound by the bait TF.

In the Y1H, a selected DNA sequence “bait” is cloned upstream of reporter genes, while the “prey” vector allows for the expression of a chimeric protein comprising a strong AD, usually the Gal4-AD, fused to either a TF of interest, a specific TF library or an entire cDNA library. TFs bearing a DBD capable of interacting with the bait sequence activate reporter gene expression [82] (Fig. 2a). Although relatively old-fashioned, the Y1H allows to identify protein-DNA interactions in vivo, thus overcoming the technical difficulties associated with recombinant TF expression/purification for in vitro assays, and to test a large number of proteins in parallel against a specific DNA element. The main disadvantages are perhaps the time-consuming step of bait-strain construction and the limit imposed by yeast transformation efficiency on the complexity of the library that can be screened (usually no more than 10^6^–10^7^ clones). Despite these limitations, a streamlined version of the Y1H interrogating 14 different bait-sequences against 988 human TF prey clones and 236 clones coding for unconventional DNA-binding proteins has allowed to identify a total of 175 DNA-protein interactions involving 13 DNA sequences and 100 TFs, including 95 transcription factors (~ 10% of the tested TFs) and five unconventional DNA-binding proteins (~ 2% of the tested proteins) [83].

Another large-scale approach for DNA-centered TF mapping relies on protein arrays. This approach was pioneered by Hu et al. who expressed, purified, arrayed and interrogated more than 4000 human proteins, and identified a number of DNA-binding proteins otherwise difficult to predict [19]. Despite their undisputable discovery potential, protein arrays remain extremely labor-intensive to produce and have not yet been integrated with advanced detection tools.

### 3.2 TF-centered approaches

Identifying the specific sequences bound by individual transcription factors can help to predict *cis*-acting regulatory modules that regulate gene expression and to elucidate gene regulatory network functioning within cells. Several well-described, and somehow standard in vitro methods can be used for determining the DNA-binding specificity of a particular transcription factor (see below). These have been recently backed up by a high-throughput heterologous method, the bacterial one-hybrid (B1H) system, whose potential advantages and pitfalls are described below.

#### 3.2.1 Bacterial one-hybrid assay

The B1H method, developed by Meng et al. [38], [84] and only requiring standard laboratory equipment, is in principle applicable to any TF that can be cloned and expressed in *E. coli*, with the advantage (similar to the Y1H) that the TF(s) of interest does not need to be expressed/purified in recombinant form (Fig. 2b and Table 1). A one-step selection is performed in bacterial cells and the size of the DNA sequence library used for TF interrogation is only limited by transformation efficiency (corresponding to 10^8^–10^9^ independent sites, i.e., 10 to 1000-fold higher than in yeast cells), which is enough to accommodate all possible combinations of 12-bp-long DNA sequences. The B1H system is built on three components: (i) a “bait” vector for the expression of the TF of interest fused to the non-essential ω-subunit of bacterial RNA polymerase, so that TF binding to a particular DNA sequence recruits RNA polymerase, thus increasing promoter activity and reporter gene expression; (ii) a “prey” vector containing a 18–28 bp randomized collection of DNA-binding sites cloned downstream to a weak promoter that drives the expression of easily selectable genes such as the heterologous yeast *HIS3* and *URA3* genes; (iii) a bacterial selection strain (US0Δ*hisB*Δ*pyrF*Δ*rpoZ*) deleted in both the *hisB* and *pyrF* genes (the bacterial homologs of *HIS3* and *URA3*) and in the gene coding for the ω-subunit of RNA polymerase. The *URA3* reporter is used for a first counter-selection step, exploiting the conversion of 5-fluoro-orotic acid (5-FOA) into the toxic base precursor 5-fluoro-uracil (5-FU) by the *URA3*-encoded, orotidine 5′-phosphate decarboxylase enzyme, which allows eliminating from the library DNA elements that drive gene reporters expression even in the absence of the heterologous TF that is being tested (“self-activating sequences”). The *HIS3* reporter is then employed to positively select bacterial cells harboring a binding site for the TF of interest by monitoring cell growth on minimal medium containing the competitive His3 inhibitor 3-amino-triazole (3-AT). Selection stringency can be varied by changing the 3-AT concentration, thus allowing the recovery of DNA-binding sites with different affinities [38], [39], [84].

B1H allowed the characterization of 84 homeodomain TFs and 35 members of the *Drosophila melanogaster* segmentation network, including Cys_2_His_2_ zinc finger, homeodomain, bHLH, bZIP, winged helix and other DNA-binding motif-containing transcription factors [39], [85]. More recently, a combination of B1H selection with HT-sequence analysis was used to determine the DNA-binding specificity of TFs. This latter approach was successfully applied to Cys_2_His_2_ zinc finger TFs encoded by the *D. melanogaster* genome and generated 94 recognition motifs spanning a total of 70 genes, plus 23 additional, alternately spliced isoforms with varied specificities [37]. In yet another B1H approach, Persikov et al. screened a large randomized Cys_2_His_2_-zinc finger library and recovered vast pools of Cys_2_His_2_ zinc fingers capable of binding a randomized DNA-binding site covering each of 64 possible 3 bp targets in two different positional contexts [86].

The main limitation of the B1H system, in addition to an as yet not proven universal performance with all eukaryotic DNA-binding motifs, is probably related to the difficulty of achieving adequately high bacterial expression levels for some eukaryotic TFs, due, for example, to differences in codon usage as well as to heterologous cellular context protein folding and toxicity problems. Accordingly, expression of only the DBD of the TF of interest has been recommended [87].

#### 3.2.2 Protein binding microarrays

Protein binding microarrays (PBMs) are a well-established, relatively fast and high-throughput microarray-based techniques for studying the binding of proteins to DNA in vitro (see also Table 1). In a typical PBM experiment, the tagged version of a known or suspected DNA-binding protein of interest is recombinantly expressed, purified and applied to a double stranded (ds) DNA microarray. This is followed by the addition of a fluorophore-conjugated anti-tag antibody to detect and quantify the amount of the protein bound to a particular DNA spot. PBMs can be built with synthetic or genome sequence-derived DNA. At present, the universal PBM is the most widely used version and is made of over 44,000 ds-oligonucleotide spots containing all possible 10 bp-long DNA-binding sites represented at least once on the array, which means that every 8 bp sequence is present on average 32 times taking into account both orientations. High-density, multi-chambered microarray platforms can test the DNA-binding ability of multiple proteins in parallel, thus allowing the HT acquisition of large data sets, e.g., for the comparative analysis of the DNA-binding specificities of related TFs. PBM analysis is highly sensitive and dynamic, allowing to resolve DNA-binding affinities that differ by less than 1.5-fold and to measure protein–DNA interactions spanning several orders of magnitude in affinity [22], [88]. By comparing the DNA-binding profiles of homologous or isoform TFs, or wild-type and mutant versions of individual TFs, it is possible to relate protein structure/sequence differences with DNA-binding specificity and/or affinity differences, thus gaining insights on the evolutionary variation of TFs and the effect of specific TF mutations in gene (dys)regulation [89].

The main limitation of the PBM approach, in addition to the need for purification of each tagged TF and the in vitro nature of the assay that may complicate a reliable extrapolation to the in vivo situation, is the inability to identify long DNA-binding sites bound by TFs with long DNA-binding motifs and/or relying on an extensive (multiprotein) network of protein–DNA interactions. In addition, some TFs may require particular post-translational modifications or protein interaction partners in order to achieve an adequate DNA-binding affinity or specificity.

Nevertheless, the PBM platform has been instrumental to a number of insightful, large-scale DNA-binding studies and to the characterization of large groups of TFs. For example, a PBM representing all the intergenic regions of the *S. cerevisiae* genome was used to map the DNA-binding sites of a large number of structurally diverse yeast TFs [27]. More recently, the PBM approach has been used to define the DNA-binding sites of 129 transcription factors representative of the major canonical TF families in *Caenorhabditis elegans*, thus allowing to infer the DNA-binding specificities for approximately 40% of the predicted *C. elegans* transcription factors [90]. The Universal PBM Resource for Oligonucleotide Binding Evaluation (UniPROBE) database (http://thebrain.bwh.harvard.edu/uniprobe) is a useful resource for universal PBM data sets derived from a range of species. This database also provides appropriate curation, easy searching and an informative display interface for universal PBM data [91].

Another microarray-based method, named Cognate Site Identifier (CSI), relies on a HT platform consisting of a ds-DNA array that displays the entire sequence space represented by 8 up to 10 variable base pair positions. The duplex DNA sequences spotted on the CSI array are self-complementary palindromes interrupted at the center by a TCCT sequence in order to facilitate hairpin DNA formation. Protein binding detection relies on chemical labeling of the TFs that are applied to the microarray, which are visualized directly (i.e., without the use of a labeled antibody). CSI array analysis was initially employed to perform a comprehensive, mutational DNA-binding site analysis in a single experiment, which provided information on the contribution of individual nucleotide residues to TF-DNA recognition [29]. An improved version of this procedure, named CSI-Fluorescence Intercalation Displacement (CSI–FID), is a plate-based technique that measures the displacement of a fluorescent dye intercalated into the DNA hairpin by an unlabeled TF in order to determine its sequence preference. By combining these technologies, it is possible to interrogate the entire sequence space of at least 10 bp-long DNA-binding sites with a high dynamic range, under label-free conditions [92].

An additional, readout-improved, variation of the PBM method is the so-called total internal reflectance fluorescence-PBM (TIRF-PBM), in which TIRF is coupled to a microarray to enable real-time detection of dye-labeled TFs binding across a microarray of immobilized DNA in hydrogels [23]. With this approach it is possible to determine both equilibrium binding specificities and kinetic rates for multiple TF:DNA interactions in a single experiment. Moreover it allows to study multi-protein complex:DNA interactions using proteins labeled with different dyes. The major drawback of this approach is its relative low throughout (limited to only ~ 100 DNA sequences at a time).

#### 3.2.3 Other HT DNA-binding site identification technologies

Besides to B1H and microarray-based techniques, many newly developed HT technologies have revolutionized the ability to characterize protein–DNA binding interactions. These additional technologies (listed in Table 1) include: Bind-n-seq [35], EMSA-seq [33], HT-SELEX/SELEX-seq [30], [32] and microarray-based investigation of genomic aptamers by shift (MEGAshift) [28]. Despite their strikingly increased throughput compared to more basic methods such as electrophoretic mobility shift assay (EMSA)- and surface plasmon resonance (SPR)-based assays, most of these techniques do not allow an accurate quantification of protein-DNA interactions and usually require complicated algorithms and associated approximations. Perhaps, the best compromise to-date between accuracy and throughput has been achieved with two techniques named mechanically induced trapping of molecular interactions (MITOMI) [36], [93], [94] and high-throughput sequencing-fluorescent ligand interaction profiling (HiTS-FLIP) [31], which allow dissociation constant determination for several transcription factors against thousands of DNA sequences (MITOMI) or a single TF against millions of DNA motifs (HiTS-FLIP).

MITOMI allows the direct, medium throughput determination of the binding affinities of individual TFs for each of a few hundred different DNA sites. Synthetic genes coding for His-tag TFs undergoing MITOMI analysis are flowed into individual chambers of a multi-chamber device along with the reagents required to support their synthesis through in vitro transcription/translation, thus avoiding possible problems associated with TF purification. Each chamber contains anti-His-tag antibodies linking the fluorescently (BODIPY-Lys) labeled TF to its surface and is seeded with a specific, fluorescently (Cy5) labeled, candidate DNA-binding sequence at a predetermined concentration. This multi-chamber assembly can thus accommodate hundreds of different DNA-binding sequences at single or multiple different concentrations.

HiTS-FLIP relies, instead, on a novel next-generation sequencing (NGS) application aimed at identifying DNA sequences bound by specific, fluorescently labeled TFs, taking advantage of the optics and fluidics of an Illumina sequencer to detect and score binding [31]. The procedure is conceptually simple, can assay up to 10^9^ protein-DNA interactions in parallel and is based on the following steps: (i) building and sequencing ~ 100 million clusters of genomic or random synthetic DNA; (ii) denature and wash away the second strand in order to rebuild ds-DNA clusters using unmodified dNTPs; (iii) introduce the fluorescently labeled query TFs into the flow cell; (iv) fluorescence-based quantification of protein binding to each DNA cluster after an optional, two-minute wash step; (v) mapping and matching the bound clusters to the corresponding sequences in order to obtain a comprehensive and quantitative DNA-binding affinity landscape.

Another well-established, TF-binding site searching procedure that has recently been integrated with a HT-NGS readout is the systematic evolution of ligands by exponential enrichment (SELEX), which employs purified TFs (or other query proteins) for the in vitro selection of high-affinity DNA-binding sites from random libraries [95], [96]. The general strategy, here, is to create DNA-binding sites libraries, derived from randomly synthesized oligos or genomic sequence fragments, containing invariant regions at both ends, to be used as primer binding sites for PCR (re)amplification after selection cycle. Purified TFs are added to the library, followed by separation of protein-bound and unbound sequences by various means such as gel-filtration, filter-binding or capturing by immobilized antibodies. This selection cycle is usually repeated 3–5 times in order to increase the fraction of captured high-affinity binding sites, followed by cloning and sequencing of the best-hits which are typically less than 100 non-redundant sequences. By coupling conventional SELEX with an NGS readout (HT-SELEX [34] or Bind-and-Seq [35]) it is now possible to obtain large-scale and comprehensive binding energy profiles. A recent HT-SELEX work [30] using unfractionated tagged proteins (rather than purified TFs) and a barcoding system for individual experiments, has further expanded the discovery potential and throughput of this approach, generating binding data for 19 different TFs.

An advantage of SELEX coupled with an NGS readout, in addition to its high feasibility, is that the final output (i.e., the number of counts/sequence) is digital, and thus guarantees an extremely broad dynamic range. Within a total set of hundreds of thousands or millions of sequences there will be many non-specific sites, which, however, usually occur only once on a statistical basis, whereas high-affinity sites may occur thousands of times. From millions of reads, even after a single round of selection, one can thus delineate a binding model (such as a PWM) and by subsequent refinement obtain the models that best fit the data [34]. The final outcome can be further improved by including data from additional rounds of selection, which may provide more accurate DNA-binding energy models even for low-specificity TFs. Another advantage of SELEX and related approaches is that there is no inherent limit to the length of the binding sites that can be screened and selected, even though the size of the library will somehow limit the length that can be covered comprehensively; for example, 1 nmol of DNA, corresponding to ~ 10^15^ non-redundant sequences, can comprise nearly all possible combinations of 25 bp-long binding sites. This makes it possible to study TFs with unusually long binding sites, including bacterial TFs, whose binding-sites are typically longer than 16 bp.

## 4 Concluding remarks

The focus of this review was on the variety of tools, both dry and wet, that can be used alone as well as integrated into different modular platforms for a functional identification and characterization of transcription factors, including the more elusive unconventional activators. In silico-based approaches represent the first step toward the creation of a comprehensive TF. Even though TF function depends on many parameters and their involvement in a specific signaling pathway is often difficult to predict (especially if only based on sequence similarity), the occurrence or lack of a particular TF can by itself point to the existence or absence of a particular pathway. Therefore, it is essential to know and compare the TF repertoires present in different species, with special reference to missing genes and to duplicated genes that may hint to a novel function.

The throughput and reliability of TF discovery approaches has greatly advanced in recent years thanks to the setting up of new HT platforms, especially microarray-based technologies and next-generation sequencing. These new approaches have been instrumental to the creation of large and detailed DNA-binding data compendia, which have facilitated TF function analysis on a genome-wide scale and also provided new insights into the molecular mechanisms underlying TF-binding specificity. This extended search also improved our understanding of the evolutionary variation of TFs and the role of particular TF mutations in causing specific gene (dys)regulation events, thus contributing to delineate a sort of TF/DBD “recognition code” [58], [89], [97], [98], [99], [100], [101], [102], [103], [104], [105], [106].

Besides conventional TFs, with their well-defined and in silico recognizable DBDs, there is a growing list of so-called “unconventional” transcriptional activators, which can be conveniently identified with various functional heterologous and homologous, yeast two-hybrid-derived screens. The interest for these moonlighting TFs is mainly related to their possible involvement in the establishment of new regulatory networks and potential implication in human diseases. Conventional and unconventional TF characterization, which heavily relies on ‘omics-based’ approaches, is thus one of the most important and productive areas of the post-genomic era.

## Figures and Tables

**Table 1:** Outline of in vitro and in vivo heterologous high-throughput DNA-binding assays^a^.

Acronym	Name	Throughput	Probe type	Resolution^b^	References
HT-SELEX	High throughput systematic evolution of ligand by exponential enrichment	10^15^	Oligo library	Qualitative (SELEX) Quantitative (HT-SELEX)	[30], [32], [34], [104]
Bind-n-Seq	Bind and sequence	10^13^	Oligo library	Quantitative	[35]
HiTS-FLIP	High throughput sequencing-fluorescent ligand interaction profiling	10^9^	Oligo library (clusters on Illumina flow cell)	Kinetics	[31]
B1H	Bacterial one-hybrid	10^8^	Oligo library (in plasmid)	Qualitative (B1H) Quantitative (B1H followed by HT-seq)	[37], [39], [84], [87]
PBM	Protein-binding microarray	10^6^	Microarray	Quantitative	[22], [24], [25], [26], [27], [90], [91], [101], [107]
CSI	Cognate site identifier	10^6^	Microarray	Quantitative	[29], [92], [108]
EMSA-seq	EMSA followed by high throughput sequencing	10^5^–10^6^	Oligo library	Quantitative	[33]
MEGAshift	Microarray evaluation of genomic aptamers by shift	10^3^	Oligo library	Quantitative	[28]
MITOMI	Mechanically induced trapping of molecular interactions	10^2^–10^3^	Oligo library	Kinetics	[36], [93], [94], [109]
HT-SPR	High throughput surface plasmon resonance	10^2^	Microarray	Kinetics	[110], [111]
TIRF-PBM	Total internal reflectance fluorescence PBM	10^2^	Microarray	Kinetics	[23], [112]

a High-throughput, TF binding site discovery approaches ordered by throughput, i.e. the approximate number of DNA sequences interrogated in parallel (as reported in the cited references); probe type refers to the specific format of the DNA probe oligomer utilized by each method.

b Qualitative: only binding sites with the highest affinity are likely to be obtained; Quantitative: binding models (e.g. PWM) can be determined; kinetics: equilibrium binding specificities and kinetic constants can be calculated.

## References

[1] Simon I., Barnett J., Hannett N., Harbison C.T., Rinaldi N.J. (2001). Serial regulation of transcriptional regulators in the yeast cell cycle. Cell.

[2] Bain G., Maandag E.C., Izon D.J., Amsen D., Kruisbeek A.M. (1994). E2A proteins are required for proper B cell development and initiation of immunoglobulin gene rearrangements. Cell.

[3] Dynlacht B.D. (1997). Regulation of transcription by proteins that control the cell cycle. Nature.

[4] Accili D., Arden K.C. (2004). FoxOs at the crossroads of cellular metabolism, differentiation, and transformation. Cell.

[5] Furney S.J., Higgins D.G., Ouzounis C.A., Lopez-Bigas N. (2006). Structural and functional properties of genes involved in human cancer. BMC Genomics.

[6] Boyadjiev S.A., Jabs E.W. (2000). Online Mendelian Inheritance in Man (OMIM) as a knowledgebase for human developmental disorders. Clin Genet.

[7] Bustamante C.D., Fledel-Alon A., Williamson S., Nielsen R., Hubisz M.T. (2005). Natural selection on protein-coding genes in the human genome. Nature.

[8] De S., Lopez-Bigas N., Teichmann S.A. (2008). Patterns of evolutionary constraints on genes in humans. BMC Evol Biol.

[9] Lopez-Bigas N., De S., Teichmann S.A. (2008). Functional protein divergence in the evolution of *Homo sapiens*. Genome Biol.

[10] Kirschner M., Gerhart J. (1998). Evolvability. Proc Natl Acad Sci U S A.

[11] Levine M., Tjian R. (2003). Transcription regulation and animal diversity. Nature.

[12] van Nimwegen E. (2003). Scaling laws in the functional content of genomes. Trends Genet.

[13] Vogel C., Chothia C. (2006). Protein family expansions and biological complexity. PLoS Comput Biol.

[14] Stegmaier P., Kel A.E., Wingender E. (2004). Systematic DNA-binding domain classification of transcription factors. Genome Inform.

[15] Luscombe N.M., Austin S.E., Berman H.M., Thornton J.M. (2000). An overview of the structures of protein-DNA complexes. Genome Biol.

[16] McCord R.P., Bulyk M.L. (2008). Functional trends in structural classes of the DNA binding domains of regulatory transcription factors. Pac Symp Biocomput.

[17] Fulton D.L., Sundararajan S., Badis G., Hughes T.R., Wasserman W.W. (2009). TFCat: the curated catalog of mouse and human transcription factors. Genome Biol.

[18] Hall D.A., Zhu H., Zhu X., Royce T., Gerstein M. (2004). Regulation of gene expression by a metabolic enzyme. Science.

[19] Hu S., Xie Z., Onishi A., Yu X., Jiang L. (2009). Profiling the human protein-DNA interactome reveals ERK2 as a transcriptional repressor of interferon signaling. Cell.

[20] Joshi R., Passner J.M., Rohs R., Jain R., Sosinsky A. (2007). Functional specificity of a Hox protein mediated by the recognition of minor groove structure. Cell.

[21] Wunderlich Z., Mirny L.A. (2009). Different gene regulation strategies revealed by analysis of binding motifs. Trends Genet.

[22] Berger M.F., Philippakis A.A., Qureshi A.M., He F.S., Estep P.W. (2006). Compact, universal DNA microarrays to comprehensively determine transcription-factor binding site specificities. Nat Biotechnol.

[23] Bonham A.J., Neumann T., Tirrell M., Reich N.O. (2009). Tracking transcription factor complexes on DNA using total internal reflectance fluorescence protein binding microarrays. Nucleic Acids Res.

[24] Bulyk M.L., Gentalen E., Lockhart D.J., Church G.M. (1999). Quantifying DNA-protein interactions by double-stranded DNA arrays. Nat Biotechnol.

[25] Field S., Udalova I., Ragoussis J. (2007). Accuracy and reproducibility of protein-DNA microarray technology. Adv Biochem Eng Biotechnol.

[26] Linnell J., Mott R., Field S., Kwiatkowski D.P., Ragoussis J. (2004). Quantitative high-throughput analysis of transcription factor binding specificities. Nucleic Acids Res.

[27] Mukherjee S., Berger M.F., Jona G., Wang X.S., Muzzey D. (2004). Rapid analysis of the DNA-binding specificities of transcription factors with DNA microarrays. Nat Genet.

[28] Tantin D., Gemberling M., Callister C., Fairbrother W.G. (2008). High-throughput biochemical analysis of in vivo location data reveals novel distinct classes of POU5F1(Oct4)/DNA complexes. Genome Res.

[29] Warren C.L., Kratochvil N.C., Hauschild K.E., Foister S., Brezinski M.L. (2006). Defining the sequence-recognition profile of DNA-binding molecules. Proc Natl Acad Sci U S A.

[30] Jolma A., Kivioja T., Toivonen J., Cheng L., Wei G. (2010). Multiplexed massively parallel SELEX for characterization of human transcription factor binding specificities. Genome Res.

[31] Nutiu R., Friedman R.C., Luo S., Khrebtukova I., Silva D. (2011). Direct measurement of DNA affinity landscapes on a high-throughput sequencing instrument. Nat Biotechnol.

[32] Slattery M., Riley T., Liu P., Abe N., Gomez-Alcala P. (2011). Cofactor binding evokes latent differences in DNA binding specificity between Hox proteins. Cell.

[33] Wong D., Teixeira A., Oikonomopoulos S., Humburg P., Lone I.N. (2011). Extensive characterization of NF-kappaB binding uncovers non-canonical motifs and advances the interpretation of genetic functional traits. Genome Biol.

[34] Zhao Y., Granas D., Stormo G.D. (2009). Inferring binding energies from selected binding sites. PLoS Comput Biol.

[35] Zykovich A., Korf I., Segal D.J. (2009). Bind-n-Seq: high-throughput analysis of *in vitro* protein-DNA interactions using massively parallel sequencing. Nucleic Acids Res.

[36] Maerkl S.J., Quake S.R. (2007). A systems approach to measuring the binding energy landscapes of transcription factors. Science.

[37] Enuameh M.S., Asriyan Y., Richards A., Christensen R.G., Hall V.L. (2013). Global analysis of *Drosophila* Cys_2_-His_2_ zinc finger proteins reveals a multitude of novel recognition motifs and binding determinants. Genome Res.

[38] Meng X., Smith R.M., Giesecke A.V., Joung J.K., Wolfe S.A. (2006). Counter-selectable marker for bacterial-based interaction trap systems. Biotechniques.

[39] Noyes M.B., Meng X., Wakabayashi A., Sinha S., Brodsky M.H. (2008). A systematic characterization of factors that regulate *Drosophila* segmentation via a bacterial one-hybrid system. Nucleic Acids Res.

[40] Reece-Hoyes J.S., Walhout A.J.M. (2012). Yeast one-hybrid assays: a historical and technical perspective. Methods.

[41] Commichau F.M., Stulke J. (2008). Trigger enzymes: bifunctional proteins active in metabolism and in controlling gene expression. Mol Microbiol.

[42] Scherrer T., Mittal N., Janga S.C., Gerber A.P. (2010). A screen for RNA-binding proteins in yeast indicates dual functions for many enzymes. PLoS One.

[43] Shi Y., Shi Y. (2004). Metabolic enzymes and coenzymes in transcription—a direct link between metabolism and transcription?. Trends Genet.

[44] Wilson D., Charoensawan V., Kummerfeld S.K., Teichmann S.A. (2008). DBD—taxonomically broad transcription factor predictions: new content and functionality. Nucleic Acids Res.

[45] Pfreundt U., James D.P., Tweedie S., Wilson D., Teichmann S.A. (2010). FlyTF: improved annotation and enhanced functionality of the *Drosophila* transcription factor database. Nucleic Acids Res.

[46] Park J., Park J., Jang S., Kim S., Kong S. (2008). FTFD: an informatics pipeline supporting phylogenomic analysis of fungal transcription factors. Bioinformatics.

[47] Gama-Castro S., Jimenez-Jacinto V., Peralta-Gil M., Santos-Zavaleta A., Penaloza-Spinola M.I. (2008). RegulonDB (version 6.0): gene regulation model of *Escherichia coli* K-12 beyond transcription, active (experimental) annotated promoters and Textpresso navigation. Nucleic Acids Res.

[48] Charoensawan V., Wilson D., Teichmann S.A. (2010). Genomic repertoires of DNA-binding transcription factors across the tree of life. Nucleic Acids Res.

[49] Vaquerizas J.M., Teichmann S.A., Luscombe N.M. (2012). How do you find transcription factors? Computational approaches to compile and annotate repertoires of regulators for any genome. Methods Mol Biol.

[50] Conesa A., Gotz S., Garcia-Gomez J.M., Terol J., Talon M. (2005). Blast2GO: a universal tool for annotation, visualization and analysis in functional genomics research. Bioinformatics.

[51] Quevillon E., Silventoinen V., Pillai S., Harte N., Mulder N. (2005). InterProScan: protein domains identifier. Nucleic Acids Res.

[52] Boyer L.A., Langer M.R., Crowley K.A., Tan S., Denu J.M. (2002). Essential role for the SANT domain in the functioning of multiple chromatin remodeling enzymes. Mol Cell.

[53] Patsialou A., Wilsker D., Moran E. (2005). DNA-binding properties of ARID family proteins. Nucleic Acids Res.

[54] Stros M., Launholt D., Grasser K.D. (2007). The HMG-box: a versatile protein domain occurring in a wide variety of DNA-binding proteins. Cell Mol Life Sci.

[55] Altschul S.F., Gish W., Miller W., Myers E.W., Lipman D.J. (1990). Basic local alignment search tool. J Mol Biol.

[56] Montanini B., Levati E., Bolchi A., Kohler A., Morin E. (2011). Genome-wide search and functional identification of transcription factors in the mycorrhizal fungus *Tuber melanosporum*. New Phytol.

[57] Reece-Hoyes J.S., Pons C., Diallo A., Mori A., Shrestha S. (2013). Extensive rewiring and complex evolutionary dynamics in a *C. elegans* multiparameter transcription factor network. Mol Cell.

[58] Weirauch M.T., Yang A., Albu M., Cote A.G., Montenegro-Montero A. (2014). Determination and inference of eukaryotic transcription factor sequence specificity. Cell.

[59] Ma J., Ptashne M. (1987). A new class of yeast transcriptional activators. Cell.

[60] Titz B., Thomas S., Rajagopala S.V., Chiba T., Ito T. (2006). Transcriptional activators in yeast. Nucleic Acids Res.

[61] Ye R., Yao Q.-H., Xu Z.-H., Xue H.-W. (2004). Development of an efficient method for the isolation of factors involved in gene transcription during rice embryo development. Plant J.

[62] Fujimoto S.Y., Ohta M., Usui A., Shinshi H., Ohme-Takagi M. (2000). *Arabidopsis* ethylene-responsive element binding factors act as transcriptional activators or repressors of GCC box-mediated gene expression. Plant Cell.

[63] Ohta M., Ohme-Takagi M., Shinshi H. (2000). Three ethylene-responsive transcription factors in tobacco with distinct transactivation functions. Plant J.

[64] Wiesner C., Hoeth M., Binder B.R., de Martin R. (2002). A functional screening assay for the isolation of transcription factors. Nucleic Acids Res.

[65] Levati E., Sartini S., Bolchi A., Ottonello S., Montanini B. (2016). Moonlighting transcriptional activation function of a fungal sulfur metabolism enzyme. Sci Rep.

[66] Kosugi S., Hasebe M., Tomita M., Yanagawa H. (2009). Systematic identification of cell cycle-dependent yeast nucleocytoplasmic shuttling proteins by prediction of composite motifs. Proc Natl Acad Sci U S A.

[67] Nguyen Ba A.N., Pogoutse A., Provart N., Moses A.M. (2009). NLStradamus: a simple hidden Markov model for nuclear localization signal prediction. BMC Bioinf.

[68] Rhee Y., Gurel F., Gafni Y., Dingwall C., Citovsky V. (2000). A genetic system for detection of protein nuclear import and export. Nat Biotechnol.

[69] Marshall K.S., Zhang Z., Curran J., Derbyshire S., Mymryk J.S. (2007). An improved genetic system for detection and analysis of protein nuclear import signals. BMC Mol Biol.

[70] Marshall K.S., Cohen M.J., Fonseca G.J., Todorovic B., King C.R. (2014). Identification and characterization of multiple conserved nuclear localization signals within adenovirus E1A. Virology.

[71] Jeziorska D.M., Jordan K.W., Vance K.W. (2009). A systems biology approach to understanding *cis*-regulatory module function. Semin Cell Dev Biol.

[72] Barski A., Zhao K. (2009). Genomic location analysis by ChIP-Seq. J Cell Biochem.

[73] Iyer V.R., Horak C.E., Scafe C.S., Botstein D., Snyder M. (2001). Genomic binding sites of the yeast cell-cycle transcription factors SBF and MBF. Nature.

[74] Park P.J. (2009). ChIP-seq: advantages and challenges of a maturing technology. Nat Rev Genet.

[75] Gordan R., Hartemink A.J., Bulyk M.L. (2009). Distinguishing direct versus indirect transcription factor-DNA interactions. Genome Res.

[76] Li X.Y., Thomas S., Sabo P.J., Eisen M.B., Stamatoyannopoulos J.A. (2011). The role of chromatin accessibility in directing the widespread, overlapping patterns of *Drosophila* transcription factor binding. Genome Biol.

[77] Liu X., Lee C.K., Granek J.A., Clarke N.D., Lieb J.D. (2006). Whole-genome comparison of Leu3 binding in vitro and in vivo reveals the importance of nucleosome occupancy in target site selection. Genome Res.

[78] Yan J., Enge M., Whitington T., Dave K., Liu J. (2013). Transcription factor binding in human cells occurs in dense clusters formed around cohesin anchor sites. Cell.

[79] Jolma A, Taipale J. Methods for analysis of transcription factor DNA-binding specificity in vitro. In A handbook of transcription factors. Edited by Hughes TR: Springer; 2011:155–173. [Harris JR (Series Editor): Subcellular Biochemistry, vol 52.] 10.1007/978-90-481-9069-0_7 21557082

[80] Slattery M., Zhou T., Yang L., Dantas Machado A.C., Gordan R. (2014). Absence of a simple code: how transcription factors read the genome. Trends Biochem Sci.

[81] Stormo G.D., Zhao Y. (2010). Determining the specificity of protein-DNA interactions. Nat Rev Genet.

[82] Deplancke B., Dupuy D., Vidal M., Walhout A.J. (2004). A gateway-compatible yeast one-hybrid system. Genome Res.

[83] Reece-Hoyes J.S., Barutcu A.R., McCord R.P., Jeong J.S., Jiang L. (2012). Yeast one-hybrid assays for gene-centered human gene regulatory network mapping. Nat Methods.

[84] Meng X., Brodsky M.H., Wolfe S.A. (2005). A bacterial one-hybrid system for determining the DNA-binding specificity of transcription factors. Nat Biotechnol.

[85] Noyes M.B., Christensen R.G., Wakabayashi A., Stormo G.D., Brodsky M.H. (2008). Analysis of homeodomain specificities allows the family-wide prediction of preferred recognition sites. Cell.

[86] Persikov A.V., Wetzel J.L., Rowland E.F., Oakes B.L., Xu D.J. (2015). A systematic survey of the Cys_2_His_2_ zinc finger DNA-binding landscape. Nucleic Acids Res.

[87] Meng X., Sa W. (2006). Identifying DNA sequences recognized by a transcription factor using a bacterial one-hybrid system. Nat Protoc.

[88] Berger M.F., Bulyk M.L. (2009). Universal protein-binding microarrays for the comprehensive characterization of the DNA-binding specificities of transcription factors. Nat Protoc.

[89] Andrilenas K.K., Penvose A., Siggers T. (2014). Using protein-binding microarrays to study transcription factor specificity: homologs, isoforms and complexes. Brief Funct Genomics.

[90] Narasimhan K., Lambert S.A., Yang A.W., Riddell J., Mnaimneh S. (2015). Mapping and analysis of *Caenorhabditis elegans* transcription factor sequence specificities. Elife.

[91] Hume M.A., Barrera L.A., Gisselbrecht S.S., Bulyk M.L. (2015). UniPROBE, update 2015: new tools and content for the online database of protein-binding microarray data on protein-DNA interactions. Nucleic Acids Res.

[92] Hauschild K.E., Stover J.S., Boger D.L., Ansari A.Z. (2009). CSI–FID: high throughput label-free detection of DNA binding molecules. Bioorg Med Chem Lett.

[93] Fordyce P.M., Gerber D., Tran D., Zheng J., Li H. (2010). De novo identification and biophysical characterization of transcription-factor binding sites with microfluidic affinity analysis. Nat Biotechnol.

[94] Maerkl S.J., Quake S.R. (2009). Experimental determination of the evolvability of a transcription factor. Proc Natl Acad Sci U S A.

[95] Oliphant A.R., Brandl C.J., Struhl K. (1989). Defining the sequence specificity of DNA-binding proteins by selecting binding sites from random-sequence oligonucleotides: analysis of yeast GCN4 protein. Mol Cell Biol.

[96] Tuerk C., Gold L. (1990). Systematic evolution of ligands by exponential enrichment: RNA ligands to bacteriophage T4 DNA polymerase. Science.

[97] Badis G., Berger M.F., Philippakis A.A., Talukder S., Gehrke A.R. (2009). Diversity and complexity in DNA recognition by transcription factors. Science.

[98] Badis G., Chan E.T., van Bakel H., Pena-Castillo L., Tillo D. (2008). A library of yeast transcription factor motifs reveals a widespread function for Rsc3 in targeting nucleosome exclusion at promoters. Mol Cell.

[99] Berger M.F., Badis G., Gehrke A.R., Talukder S., Philippakis A.A. (2008). Variation in homeodomain DNA binding revealed by high-resolution analysis of sequence preferences. Cell.

[100] De Silva E.K., Gehrke A.R., Olszewski K., Leon I., Chahal J.S. (2008). Specific DNA-binding by apicomplexan AP2 transcription factors. Proc Natl Acad Sci U S A.

[101] Franco-Zorrilla J.M., Lopez-Vidriero I., Carrasco J.L., Godoy M., Vera P. (2014). DNA-binding specificities of plant transcription factors and their potential to define target genes. Proc Natl Acad Sci U S A.

[102] Gordan R., Murphy K.F., McCord R.P., Zhu C., Vedenko A. (2011). Curated collection of yeast transcription factor DNA binding specificity data reveals novel structural and gene regulatory insights. Genome Biol.

[103] Grove C.A., Masi F.D., Barrasa M.I., Newburger D.E., Alkema M.J. (2009). A multiparameter network reveals extensive divergence between *C**. elegans* bHLH transcription factors. Cell.

[104] Jolma A., Yan J., Whitington T., Toivonen J., Nitta K.R. (2013). DNA-binding specificities of human transcription factors. Cell.

[105] Wei G.H., Badis G., Berger M.F., Kivioja T., Palin K. (2010). Genome-wide analysis of ETS-family DNA-binding in vitro and in vivo. EMBO J.

[106] Zhu C., Byers K.J., McCord R.P., Shi Z., Berger M.F. (2009). High-resolution DNA-binding specificity analysis of yeast transcription factors. Genome Res.

[107] Lindemose S., Jensen M.K., Van de Velde J., O'Shea C., Heyndrickx K.S. (2014). A DNA-binding-site landscape and regulatory network analysis for NAC transcription factors in *Arabidopsis thaliana*. Nucleic Acids Res.

[108] Puckett J.W., Muzikar K.A., Tietjen J., Warren C.L., Ansari A.Z. (2007). Quantitative microarray profiling of DNA-binding molecules. J Am Chem Soc.

[109] Geertz M., Shore D., Maerkl S.J. (2012). Massively parallel measurements of molecular interaction kinetics on a microfluidic platform. Proc Natl Acad Sci U S A.

[110] Campbell C.T., Kim G. (2007). SPR microscopy and its applications to high-throughput analyses of biomolecular binding events and their kinetics. Biomaterials.

[111] Shumaker-Parry J.S., Aebersold R., Campbell C.T. (2004). Parallel, quantitative measurement of protein binding to a 120-element double-stranded DNA array in real time using surface plasmon resonance microscopy. Anal Chem.

[112] Bonham A.J., Wenta N., Osslund L.M., Prussin A.J., Vinkemeier U. (2013). STAT1:DNA sequence-dependent binding modulation by phosphorylation, protein:protein interactions and small-molecule inhibition. Nucleic Acids Res.

